# A Concise History of the Principal Discoveries in Anatomy

**Published:** 1800-03

**Authors:** 


### *A Concise History of the Principal Discoveries in Anatomy.*

[Continued from our last Number, pp. 156—161.

§. 32. THE knowledge of the structure of the brain, and the distribution of the nerves to different parts of the body, was considerably improved during the sixteenth century; although the doctrine of their functions still savoured of the old Galenian Theory. Thus, the *spiritus animales* originated in the ventricles of the brain; and thus the blood, mixed with the vital fluid, was evacuated from the arteries through the tortuous windings of the brain into those ventricles, in order that the animal spirits might thence be secreted. This was the doctrine doctrine of BERENGAR,* as well as that of most physiologists of his time. It is likewise remarkable, that the superficial parts of the brain were understood only at a very late period; while the parts situated below the basis of the ventricles, were more minutely examined. The reason of this apparent inconsistency is probably to be found in the less striking appearance of the former, as well as in the commonly received opinion, that the ventricles constituted the principal parts of the brain, and consequently ought to be first examined. BERENGAR had the knowledge of four ventricles, and described at the base of the two first, or the lateral ventricles, the *plexus choroideus*, by the appellation of vermiform processes, together with the substance formed of arterial and venous vessels; † he represented the canal which connects the fourth ventricle in the medulla oblongata with the lateral ventricles, ‡ the *eminentiæ candicantes*, behind the thalami nervorum opticorum, on the base of the cranium, and likewise the pineal gland. § The abovementioned canal in the substance of the corpora quadrigemina was, however, denominated *aquæductus Sylvii*, in commemoration of that author, who first distinctly described it. ‖ Subsequent to SYLVIUS, VESALIUS gave an accurate account of this canal, which was by him called an aqueduct. ¶ He besides observed two lamellæ in the dura mater, which had been previously discovered by MASSA.** He was acquainted with the cineritious cortical substance, which he distinguished from the medullary; †† he also described the *ventriculus tricornis* with more exactness than his predecessors, and refuted the prevailing opinion, that the sense of smelling was situated in its anterior cornua. He farther proved that the ventricles were not furnished internally with any peculiar membrane, and endeavoured to explain that their use consisted merely in preserving and supporting the animal spirits. He lastly described the choroid plexus, at the base of the ventricles, where he discovered two new parts, the *septum lucidum* and the *fornix*. ‡‡

SERVETUS, in his work before quoted, applied the discoveries of VESALIUS to establish his hypothesis of the animal functions: he believed that the plexus choroideus served for secerning the animal spirits; §§ but that the real seat of the soul was situated in the acqueduct discovered by SYLVIUS; ‖‖ the

* Commentar. in *Mundin*. f. 431. a.

† Comment. in Mundin. f. 437. a.

§ *Ib*. f. 437. a. 442. a.

‖ Dissect. 4. p. 310.

¶ Lib. vii. c. 6. p. 546.

** Introduct. f. 83. b.

†† Lib. vii. c. 4, p. 541.

‡‡ Lib. vii. c. 7. p. 547.

§§ (*Servet.*) restitut. christian. lib. v. p. 171.

‖‖ *Ib*. p. 175.

two anterior ventricles represented the images of exterior objects; the third was the seat of thought, and the fourth that of memory.*

§ 33. In the tables of EUSTACHIUS, there occurs a very good figure of the *basis cerebri*, and some other representations of the interior parts of the brain, † which do credit to those times.

ARANZI discovered, soon after EUSTACHIUS, the *pedes hippocampi*, at the sides of the posterior parts of the *corpus callosum*, ‡ and described also the fourth ventricle, by the name of *cisterna cerebelli*, § as his own discovery. VAROLI represented, with more precision than any of his predecessors, the *commissura anterior et posterior*, the *crura medullæ oblongatæ*, ‖ the bridge of his denomination, ¶ and the choroid plexus of the brain, as consisting of a glandular substance: ** he, however, did not clearly comprehend the connection of the fourth with the first ventricle. †† At length, PICCOLHUOMINI distinguished, in the most accurate manner, next to VESALIUS, the cineritious cortical from the medullary substance of the brain. ‡‡

With respect to the medulla spinalis, ACHILLINI was not ignorant that its substance terminated in the region of the loins; §§ but BERENGAR more exactly described its termination towards the twelfth vertebra of the back, in a small oval plexus. ‖‖ STEPHANUS ¶¶ and PICCOLHUOMINI *** inform us, that a cavity is sometimes observed in the spinal marrow, replete with a yellow substance; an observation which is confirmed by a celebrated modern anatomist. †††

BAUHINUS appears to have been acquainted with the *ligamentum serratum*, which connects the pia with the dura mater of the spinal marrow. ‡‡‡

§ 34. With the exception of the followers of ARISTOTLE, and especially of CÆSALPINUS, all other authors of this century derived the nerves from the brain. But CÆSALPINUS spared no pains in supporting the hypothesis of ARISTOTLE, that the heart was the origin of the nerves, by asserting, that in the living body there could be but one principal part, and one

---

* *(Servet.)* restitut. christian. lib. v. p. 177.

† Tab. xvii. xviii.

‡ Obs. c. 1. p. 43.

§ *Ib.* c. 7. p. 48.

‖ De nerv. opt. f. 3. b.

¶ *Ib.* f. 4. a.

** *Ib.* f. 8. a.

†† *Ib.* f. 6. b.

‡‡ Anatom. praelect. p. 252.

§§ Annotat. in Mundin. p. 45.

‖‖ Comment. in Mundin, f. 496. b.

¶¶ *Stephan.* de dissect. p. 337.

*** Anatom. praelect. p. 260.

††† *Morgagni* adversar. anatom. vi. animadv. 14. p. 17. 18.

‡‡‡ Theatr. lib. iii. tab. xv. fig. 1.—Compare *Haller*. element. physiol. vol. iv. p. 86.

one reſidence of the mind, there being but one ſoul. As the heart is the firſt viſible object in the punctum ſaliens of the fecundated egg, it muſt alſo be the pre-eminent part of the body, and the only reſidence of the ſoul; and, if this be admitted, the heart muſt conſequently be the ſenſorium commune, and likewiſe the origin of the nerves, as is evident from the effect of the paſſions on the heart. And although ocular inſpection, as well as daily experience, ſufficiently evince that the nervous influence originates in the brain, yet CÆSALPINUS, in admitting the theory of ARISTOTLE, defended himſelf by aſſerting, that the arteries originally tranſmitted the nervous energy from the heart to the brain, and were already furniſhed with nervous membranes, their cavities collapſing within the brain, and the membranes attenuating into filaments, where they changed into nerves.* Few anatomiſts of that age coincided in opinion with CÆSALPINUS; nevertheleſs, the obſervations made on the inſenſibility of the cortical ſubſtance, ſeemed in ſome reſpect to favour his conjecture. † With a view to invalidate this atteſted fact, VAROLI objected, though not in a ſatisfactory manner, that the brain being the ſeat of all the organs of ſenſe, cannot manifeſt a greater ſuſceptibility of feeling for the one than for the other organ of ſenſation.‡

The opinions concerning the origin of the nerves from the *cerebellum*, were ſtill more divided. GALEN had maintained, that the nerves of a harder conſiſtence originated from the cerebellum; but BERENGAR aſſerted the contrary, and alleged a number of experiments purpoſely inſtituted, by which he was convinced that the cerebellum emitted no nerves whatever, but all, without exception, owed their origin to the cerebrum, or the medulla oblongata. § COLUMBUS alſo ſeriouſly maintained this opinion; ‖ but VAROLI proved that the medullary proceſſes of the cerebellum contributed to form the ſpinal marrow, and that the auditory nerves were emitted from the pores Varolii, or from the medullary nodus itſelf, as this is an important part of the cerebellum. ¶ This ſubject is now leſs contended, and the acouſtic nerve is generally ſuppoſed to ariſe from the corpora pyramidalia; yet the facial, as well as the fifth and ſixth pair of nerves, generally originate either from the pores Varolii, or the nodus medullaris. The old prejudice, that the nerves originated partly from the *meninges cerebri*, or, at leaſt, that all the nerves were ſurrounded with the two membranes

* *Caeſalpin.* quaeſt. peripatet. lib. v. c. 3. p. 514. f.

† *Laurent.* hiſt. anatom. lib. x. quaeſt. 9. p. 857.

‡ *Varol.* anatom. lib. i. c. 3. p. 6. § Comment. in Mundin. f. 434. a.

‖ Lib. viii. c. p. 356. ¶ *Varol.* de nerv opt. f. 3. b.

of the brain, was firſt corrected by FALLOPIUS, who demonſtrated that the optic nerve alone was covered by the dura mater. * The diſtinction between ſenſorial and motorial nerves, † the conſequence of this hypotheſis, was, on more accurate examination, found to be erroneous. DU LAURENS evinced that the par vagum, (which he denominated the ſixth pair) ſerved the purpoſes both of ſenſation and motion, and that all the ſoft nerves did not produce ſenſation, nor all the hard ones motion. ‡ STEPHANUS, however, believed that the nerves proceeding to the muſcles were provided with a firm coat, and that they changed their conſiſtence from a medullary to a membranous form. §

FALLOPIUS juſtly remarked, that the diviſion of nerves into pairs, according to the foramina exiſting in the cranium, was improper, becauſe two or more nerves of a diſtinct origin frequently paſs through the ſame foramen. ‖ He alſo was the firſt, ſince the time of GALEN, who diſcovered the *ganglia* of the nerves. On deſcribing his ſixth pair, (according to our diviſion, the tenth, or par vagum) he alſo mentions the ſuperior or corpus olivare, formed of the intercoſtal and the firſt cervical nerve. ¶ FALLOPIUS ſuppoſed that this ganglion belonged to the par vagum, becauſe, in his opinion, the intercoſtal nerve originated from the par vagum; and, indeed this ganglion often receives branches from the tenth pair. **

§ 35. We ſhall now treat upon the individual nerves, in order to aſcertain the relative degree of knowledge poſſeſſed by the anatomiſts of that century, with regard to the diſtinction of the nerves. In the deſcription of the firſt pair, or the olfactory nerve, there remains but little to add to the hiſtory already given by the worthy and celebrated METZGER. †† It is evident that anatomiſts were altogether unacquainted with the olfactory nerve, at the commencement of the ſixteenth century; they merely treated of mammillary proceſſes of the brain, or papillæ too ſoft and tender to be regiſtered among the nerves, and the uſe of which was to ſecrete the viſcid humours from the cerebral ventricles, and receive the odorous effluvia. Such was the deſcription of ZERBIUS, ‡‡ who thus gave a farther proof that he

* *Fallop.* obſ. p. 402.

† Compare *Prof. Sprengel's Hiſtory of Medicine,* Vol. I. p. 385.

‡ *Laurent* hiſt. anat. lib. iv. quaeſt. 10. p. 292,

§ *Stephan.* de diſſect. p. 9●.

‖ *Fallop.* obſ. p. 403.

¶ *Ib.* p. 407.

** *Neubauer* de nerv. cardiac. tab. iii. fig. 1. nr. 11.

†† *Metzger* primi paris nervorum hiſtoria in *Ej.* opuſc. anat. et phyſiolog. 8. Goth. et Amſteld. 1790, and in *Ludwig* ſcript. nevrolog. minor. vol. i. p. 108. f.

‡‡ *Zerb.* anatom. lib. iv. p. 123. 124.

he did not conſider theſe parts as nerves, ſince he begun the enumeration of the particular nerves from the optic. PORTAL* and HALLER † are therefore wrong in ſtating, that ZERBIUS already knew the olfactory nerve. ACHILLINI, to whom METZGER had no acceſs, ſpeaks evidently of the diſtribution of the olfactory nerve to the noſe, and of its progreſs under the papillæ, from which he conſequently diſtinguiſhes the nerve itſelf. He complains, however, that he has frequently been unable to find this nerve,‡ which SOEMMERING juſtly aſcribes to its ſudden putrefaction as it is very tender, and only diſcernible in freſh ſubjects. § Should therefore ACHILLINI not be regarded as the *diſcoverer of the olfactory nerve?* When SOEMMERING and METZGER ſhall have read ACHILLINI, it is to be hoped they will anſwer this queſtion in the affirmative. BERENGAR, || on the contrary, and WINTHER VON ANDERNACH, ¶ knew no more, than that theſe papillary proceſſes, though real organs of ſcent, were no nerves; that they terminated in the os cribriforme, and admitted through its holes, a liquor, ſecreted into the noſe. Even MASSA treated on the origin of this nerve with no ſmall ingenuity; he attributed to it all the properties of a nerve, deſcribed its extenſion to the olfactory membrane, and denominated it the firſt pair. ** And though aided by ſuch a predeceſſor, VESALIUS adopted the deſcription of what the ancient authors called papillæ; contending, that the proceſſes of theſe parts did not extend beyond the brain, and thus excluding them from the liſt of the nerves. †† He obſerved, however, the ſubtile, dewy liquor, ſecreted in young perſons from the tender coat of the olfactory nerve, collateral to the criſta galli. ‡‡ INGRASSIAS traced this nerve through the foramina cribroſa, but does not mention its further extenſion to the membrana Schneideriana. §§ Nor did COLUMBUS, |||| FALLOPIUS, ¶¶ and STEPHANUS, make much greater progreſs, the laſt of whom mentions ſome canals or pores diſcovered by him, in the year 1545. ***

[To be continued in our next Number.]

---

* Hiſt. de l'anatom. vol. 1. p. 253.

† Element. phyſiol. vol. iv. p. 205.

‡ Annot. in Mundin. p. 14. ſ.

§ De baſi encephali, lib. iii. ſ. 1. ſect. 23.

|| Comment. in Mundin. f. 450. a. b.

¶ Inſtit. anatom. p. 116.

** Introduct. f. 87.—Epiſt. medic. 6. f. 58. b.

†† Lib. iv. c. 3. p. 364. The roots of the olfactory nerve are likewiſe repreſented in a very indifferent ſtyle. See his fig. i. f. p. 362.

‡‡ Lib. vii. c. 3. p. 539.—Compare *Pfeffinger* de ſtructur. nervor. ſect. 2. §. 3.

§§ Comment. in *Galen.* de oſſib. p. 103.

|||| Lib. viii. c. 2. p. 358.

¶¶ *Fallop.* obſ. p. 402.

*** *Stephan.* de diſſect. p. 255.

